# Δ^3^,2-Hydroxybakuchiol Attenuates Depression in Multiple Rodent Models Possibly by Inhibition of Monoamine Transporters in Brain

**DOI:** 10.1155/2018/1325141

**Published:** 2018-06-20

**Authors:** Gang Zhao, Li-he Guo, Wei Huang, Jia-liang Hu

**Affiliations:** ^1^Internal Division of Traditional Chinese Medicine, Dahua Hospital, Xuhui District, Shanghai 200237, China; ^2^Teaching Hospital of Jiangsu University, Xuhui District, Shanghai 200237, China; ^3^Institute of Biochemistry and Cell Biology, Shanghai Institutes for Biological Sciences, Chinese Academy of Sciences, Shanghai 200031, China; ^4^Engineering Research Center of Modern Preparation Technology of Traditional Chinese Medicine, Ministry of Education, Shanghai University of Traditional Chinese Medicine, Shanghai 201203, China

## Abstract

Δ^3^,2-Hydroxybakuchiol is isolated from* Psoralea corylifolia (L.)*, which has therapeutic applications in traditional Chinese medicine. Our previous studies have showed that Δ^3^,2-hydroxybakuchiol inhibited the decreased activity of reserpinized mice, suggestive of its antidepressive potential. In this study, we explored the antidepressant profile of Δ^3^,2-hydroxybakuchiol in various rodent models and its possible monoamine-modulating mechanism. Δ^3^,2-Hydroxybakuchiol significantly reduced immobility time of mice in forced swim test and tail suspension test. Δ^3^,2-Hydroxybakuchiol also significantly increased sucrose consumption in chronic unpredictable mild stress (CUMS) rat model. Furthermore, isotope uptake study showed that Δ^3^,2-hydroxybakuchiol inhibited the activity of human dopamine transporter (DAT) and norepinephrine transporter (NET) in transporter-overexpressing pheochromocytoma (PC12) cells with IC_50_ values similar to the potency of bupropion. Microdialysis showed that Δ^3^,2-hydroxybakuchiol increased dopamine and norepinephrine concentration in rat striatum. In summary, Δ^3^,2-hydroxybakuchiol exerts antidepressant effects on various types of depression models through a possible mechanism of monoamine transporter inhibition.

## 1. Introduction

Depression is a mental illness characterized by significant and persistent sadness and sometimes irritability, which is predicted to be the greatest health killer worldwide in 2030 according to reports by WHO [1]. Symptoms of depression include pleasure loss, hopelessness, energy lack, slow movements, and even suicidal thoughts. Depression intervention has emerged as one of the hot topics in medicine field [2]. Despite clinic application of several antidepressant drugs for recent years, antidepressants with high-efficiency and low-toxicity still need to be developed. Depression is known as mental disorder with monoamine imbalances in brain subregions like frontal lobe, hippocampus, etc. [3], which, up to now, has become one of well-known pathogeneses for this disease. Numerous studies have demonstrated that monoamine transporters play a role in monoamine reuptake back into neuronal cell body and then lowering levels of monoamine in synaptic cleft in brain [4]. These transporters have, thus, been recognized as important targets for retaining monoamine neurotransmitters in synaptic cleft and then as targets for antidepressant drug development [2].

**Figure 1:**
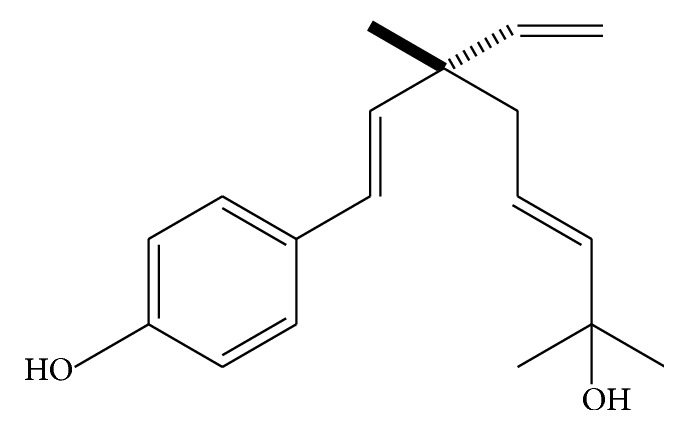
Two-dimensional representation of Δ^3^,2-hydroxybakuchiol.

In our previous study, Δ^3^,2-hydroxybakuchiol (Figure 1), isolated from Fructus Psoraleae, fruit of* Psoralea corylifolia* (L.), which was documented in ancient Chinese medical books as one of mental disorder therapeutic herbs [5], was confirmed as an active compound that acted as monoamine reuptake inhibitor (potent for DA/NE uptake and mild for 5-HT uptake) in rodent CHO cell line overexpressing rat dopamine transporter (DAT), norepinephrine transporter (NET), or serotonin transporter (SERT) [6], preliminarily demonstrating that it may be trimonoamine transporter inhibitor. In recent decades, trimonoamine transporter antagonist is thought to represent high clinic effectiveness in development of antidepressants [2]. Additionally, in our previous* in vivo* study, Δ^3^,2-hydroxybakuchiol was corroborated to protect reserpinized mice from activity decrease [6], which is known as one of the depression symptoms by some experts. It is suggested that, from the above-mentioned facts, Δ^3^,2-hydroxybakuchiol could possess an antidepressant potential. Considering key role of monoamine transporters in depression pathogenesis and the antidepressant potential of Δ^3^,2-hydroxybakuchiol, Δ^3^,2-hydroxybakuchiol may be an important candidate for antidepressant development.

In present study, we evaluated whether Δ^3^,2-hydroxybakuchiol could possess antidepressant action by using acute or chronic rodent models and whether this action could be related to monoamine modulation.

## 2. Materials and Methods

### 2.1. Reagents, Lentiviruses, and Cells

Bupropion was purchased from Aventis Pharma Co., Ltd. (Cat No. E8875, Hainan, China). Cocaine was purchased from China Food and Drug Administration. Δ^3^,2-Hydroxybakuchiol was extracted and isolated from* Psoralea corylifolia (L.)* as described in our previous publication [6] with 96.54% of purity, analyzed by high performance liquid chromatography (HPLC) detection. A voucher specimen (Zhao, 9666) has been deposited in Shanghai University of Traditional Chinese Medicine. Bupropion, cocaine, or Δ^3^,2-hydroxybakuchiol was dissolved in normal saline containing 1% dimethyl sulfoxide (DMSO) when used. Lentiviruses encoding human DAT or human NET were obtained commercially with puromycin phosphotransferase selection gene (Genepharma, China). Pheochromocytoma (PC12) and Michigan Cancer Foundation-7 (MCF-7) cell lines were purchased from Institute of Biochemistry and Cell Biology, Shanghai Institutes for Biological Sciences.

### 2.2. Animals

BALB/c mice and Sprague-Dawley rats, male and adult, were both purchased from Shanghai SIPPR-B&K Laboratory Animal Corp., Ltd. (certificate number: 856-425130, Shanghai, China). The mice and rats were maintained at room temperature (23±1°C) with 12 h light/dark alternation and free access to water and chow, except tests. All animal care and procedures were conducted according to the protocols and guidelines approved by Laboratory Animal Center of Chinese Academy of Science for the use of animal subjects.

### 2.3. Methods

#### 2.3.1. Tail Suspension Test (TST)

For TST, an acute model used for assessing antidepressant activity [7], the mice were randomly divided into 5 groups with 10 mice in each group: vehicle group treated intraperitoneally (i.p.) by injection with normal saline containing 1% DMSO, BR2.5 group (with 2.5 mg/kg bupropion as positive control), and BU2.5, BU10, and BU40 groups (with 2.5, 10, and 40 mg/kg Δ^3^,2-hydroxybakuchiol, respectively). TST was performed as described in literature [8]. After 30 min of intraperitoneal injection of vehicle or drug into mice, each mouse was hung by fixing its tail 15 cm from the ground for 6 min. Because the mice were in an abnormal position, they tried to escape and reach for ground but showed intermittent immobility during this time. The immobility time during the last 4 min was recorded.

#### 2.3.2. Forced Swim Test (FST)

FST, another model for testing acute depression profile [9], was employed to detect the antidepressant-like effect of Δ^3^,2-hydroxybakuchiol. Mice were divided into 5 groups and treated with vehicle or drugs as described in TST. FST procedure was performed as described in literature [8] with minor modification. The mice were placed in a round glass container with dimensions (diameter ×height) of 18 cm×18 cm. The water was 10 cm deep, 25°C. The experiment was conducted 30 min after drug treatment. The immobility time of each mouse during the last 4 min of 6 min test was recorded. The criteria for animal immobility recognition were as follows: (1) mice stopped struggling; (2) mice were floating; or (3) mice made occasional body movements to keep their head above the water. The immobility period of each mouse was recorded by a forced swimming operating system from Geelong software company (Australia).

#### 2.3.3. Open Field Test (OFT)

OFT was used to measure locomotor activity and behavioural interest [10]. OFT was performed as described in Ding's report [11]. Briefly, the mice were placed in centre of an opaque black box with dimensions (length×height×width) of 60 cm×40 cm×25cm; bottom of the box was marked with 10 cm×10 cm squares. Mice were habituated in cages in test room for 60 min and then divided into 5 groups: vehicle, cocaine, bupropion, BU10, and BU40 group. After treatment in each group (i.p.) with vehicle solution, 15 mg/kg cocaine, 2.5 mg/kg bupropion group, and 10 mg/kg or 40 mg/kg Δ^3^,2-hydroxybakuchiol, respectively, a 45-min measurement was conducted. Line breaks of each mouse per 15 min were recorded by a camera (Shanghai Ji Software Co., Ltd., China). Line breaks were counted by the same technician from video in computer.

#### 2.3.4. Sucrose Consumption Test (SCT)

SCT was conducted by using a chronic unpredictable mild stress (CUMS) model to evaluate drug protection of rats stressed by various stimulations against anhedonia [12]. Firstly, rats were randomly divided into 5 groups with n=10: Non-CUMS/Vehicle group with vehicle solvent treatment but no CUMS stimulation, CUMS/Vehicle group with vehicle solvent treatment, 2.5 mg/kg bupropion group (BR2.5) used as positive control, or 2.5 mg/kg and 10 mg/kg Δ^3^,2-hydroxybakuchiol group (BU2.5 and BU10, respectively) (all drug groups with corresponding dosage drug mentioned above). Except for Non-CUMS/Vehicle group, each rat in the other four groups was fed in a single cage and made to gradually develop anhedonia with a series of CUMS events. CUMS procedure was as follows: soiled bedding (200 ml of water in saw dust per cage) for 24 h, sand cushion for 24 h, empty cage for 24 h, cold stimulation (i.e., swimming in cold water at 4°C) for 5min, heat stimulation (in a hot oven at 45°C) for 5 min, cage tilt (45 degrees) overnight, 1 mA electrical stimulation plus periodic noise (10 dB) for 5 min, overnight illumination, fasting for 24 h, and water deprivation for 24 h. All stimuli were randomly applied to one type in one day and ensured that they did not occur at the same time. CUMS were conducted for continuous 8 weeks. After the first four weeks, CUMS rats were then i.p. treated with corresponding above-mentioned vehicle or drug once a day for another four weeks. During the last four-week treatment, all rats were made to experience SCT to evaluate anhedonic-like behaviour. Briefly, before CUMS procedure on the first day of each week, the rats were deprived of water for 18 h, followed by free access to two bottles each containing 100 ml of 1% sucrose water or 100 ml of distilled water. After one-hour fluid intake, initial and final weights of sucrose bottle were recorded and then consumption of sucrose water was calculated.

#### 2.3.5. Transfection of Human DAT and NET into PC12 Cells and Cell Culture

To present a verification of inhibitory effect of Δ^3^,2-hydroxybakuchiol on human DAT and NET, pheochromocytoma cell line PC12 highly expressing human DAT or human NET was stably constructed. Briefly, PC12 cells were infected with lentiviruses encoding human* DAT* or human* NET*. Using puromycin resistance, infected PC12 cells were subcloned by limiting dilution method to acquire the cells which stably expressed high level of human DAT or NET. Then, using monoamine uptake assays, we chose subclone with highest uptake activity that showed the biggest uptake difference between subclones infected with target genes and those with empty vector. The selected subclones were defined as PC12/hDAT and PC12/hNET.

#### 2.3.6. Assay for DA/NE Uptake

First, 6×10^4^ PC12/hDAT or PC12/hNET cells were seeded into each well of 96-well plates in DMEM containing 10% foetal bovine serum. After overnight incubation, culture medium was discarded, the cells were washed with phosphate-buffered saline (PBS) three times, and then 80 *μ*l/well Hank's balanced salt solution (HBSS) was added. The cells were then preincubated for 10 min at 25°C. Then, [^3^H] DA or [^3^H] NE**(**Amersham Pharmacia Biotech, USA), ascorbic acid, and pargyline were added with volume of 100 *μ*l in each well. The cells were then incubated for another 20 min at 37°C, followed by washing three times with ice-cold PBS to terminate the reaction. Subsequently, lysis buffer (2 N NaOH) was added to each well, and then an equal volume of lysate from each well was transferred to 1.2 ml of scintillation liquid. Then isotope content representing monoamine uptake was measured by a liquid scintillation counter (Beckman LS 5000TA) and recorded disintegrations per minute (DPM) (isotope content) by a liquid scintillation counter (Beckman LS 5000TA). The final concentrations of [^3^H]DA (8.8 Ci/mmol), [^3^H]NE (40 Ci/mmol), ascorbic acid, and pargyline were 100 nM, 25 nM, 100 *μ*M, and 100 *μ*M, respectively. For Δ^3^,2-hydroxybakuchiol activity evaluation, serial concentrations of Δ^3^,2-hydroxybakuchiol were added in the reaction system. Uptake inhibitory potency was measured by 50% inhibitory concentration (IC_50_) values which was analyzed by concentration-inhibition (%) curves using 4-PL formula of nonlinear aggression.

#### 2.3.7. Microdialysis

*(1) Microdialysis Probe Implantation*. The utilized dialysis probes in rats were concentric microdialysis probes (500 *μ*m diameter, 4 mm length) with fiber membranes to cut off the proteins more than 13 kDa (Spectra/Por RC, Spectrum Laboratories Inc., CA). With experimentation, rats were anesthetized with 400 mg/kg chloral hydrate. By an automated micropositioner, the speed of probes lowering into rat brain is 5 *μ*m/s. Implant position of probes is in striatum at the following coordinates: +0.0 mm anteriorly, +3.0 mm lateral to bregma, and -6.0 mm ventral from dura, with incisor bar set at -2.4 mm below intra-aural line. Then probes were fixed permanently to skull by bone-screws and acrylic cement. After surgery, rats were allowed to recover for 48 h with free access to water and chow in single cage. 

*(2) Experimental Design and In Vivo Microdialysis*. Two freely moving rats were used to collect baseline sample. Three rats were intraperitoneally injected with 10 mg/kg Δ^3^,2-hydroxybakuchiol (dissolved in PBS containing 1%DMSO, 100 *μ*M vitamin C, and 100 *μ*M pargyline). For each rat experiment, inlet tubing was connected to a syringe, which was placed in a pump at a flow rate of 1 *μ*l/min, and probe was perfused with artificial cerebrospinal fluid (aCSF). Dialysate fractions were collected into collection microtubes placed in ice at 15 min interval for continuous 120 min. The samples were stored in ice and instantly sent for HPLC detection. 

*(3) HPLC and Measurement of Monoamine Neurotransmitters*. A liquid chromatographic system was applied to measure concentrations of monoamine neurotransmitters 5-HT, DA, and NE in microdialysis samples. The samples were centrifuged at 4,000 g and 4°C for 10 min and supernatants were collected. 30 *μ*l of microdialysate was prediluted with 10 *μ*l of internal label working solution. Sample injection volume was 20 *μ*l for analysis. HPLC column of Agilent Eclipse XDB-C18 (4.6mm × 150mm, 5 *μ*m) was applied with mobile phase A (pH 5.4) of 12 g KH_2_PO_4_, 0.177g NaCl, and 0.014g EDTA and mobile phase B of methyl alcohol. The gradient elution is 0 min~12 min, 4%~4% of B; 12 min~14 min, 4%~16% of B; 14 min~29 min, 16%~16% of B; 29 min~30 min, 16%~4% of B. The analysis conditions were as follows: 0.6 ml/min of flow rate, 35°C of column temperature, and 800 mV of electrical potential. The calculated neurotransmitter concentration in dialysate was expressed as *μ*M.

#### 2.3.8. Tumourigenicity Test

The breast cancer cell line, MCF-7, was seeded in 96-well plates at 4 × 10^3^ cells per well. Then, 10 *μ*l of HBSS containing 1% DMSO or different concentrations of compound (Δ^3^,2-hydroxybakuchiol or 17-*β* estradiol) were added to control group or experimental group wells, respectively. After continuous culture for 72 h, 3-(4,5-dimethylthiazol-2-yl)-2,3-diphenyltetrazolium bromide (MTT) was added to wells at a final concentration of 0.5 mg/ml. After 4 h incubation at 37°C the liquid in plate was discarded, and 100 *μ*l/well DMSO was added. The plate was incubated for approximately 10 min with shaking. Optical density (OD) value was then measured with a microplate reader at 490 nm.

#### 2.3.9. Data Statistics

The data were presented as mean ± standard deviation and processed using SPSS13.0 software. One-way ANOVA was used followed by post hoc test for group difference. For sucrose consumption test, differences between the Non-CUMS/Vehicle and CUMS/Vehicle group were evaluated using the unpaired* t*-test.* P* < 0.05 was considered to indicate statistical significance.

## 3. Results

### 3.1. Effect of Δ^3^,2-Hydroxybakuchiol in TST

**Figure 2:**
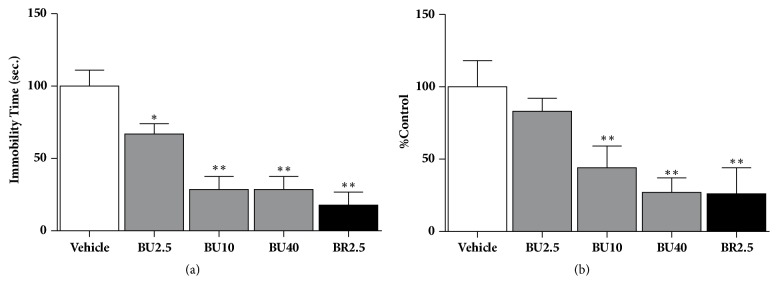
Δ^3^,2-hydroxybakuchiol administration decreased the immobility time in the mouse subjected to FST and TST. The tests were performed after male Balb/c mice received 30 min intraperitoneal injections of vehicle solvent, 2.5 mg/kg bupropion, and 2.5, 10, and 40 mg/kg Δ^3^,2-hydroxybakuchiol, respectively (n =10). (a) Immobility time in TST. (b) Percentage of immobility time (% vehicle) in FST. The data are expressed as mean ± SEM. Compared with vehicle, *∗P* < 0.05 and *∗∗P* < 0.01.

Figure 2(a) showed antidepressant effect of Δ^3^,2-hydroxybakuchiol on immobility time of the mice in TST. When injecting mice at doses of 2.5, 10, and 40 mg/kg, Δ^3^,2-hydroxybakuchiol significantly reduced immobility time in TST (*P <* 0.05 or 0.01, compared to control group). The active dosage of 10 or 40 mg/kg Δ^3^,2-hydroxybakuchiol produced an action similar to that of 2.5 mg/kg bupropion (*P >*0.05, 10 or 40 mg/kg Δ^3^,2-hydroxybakuchiol versus 2.5 mg/kg bupropion).

### 3.2. Effect of Δ^3^,2-Hydroxybakuchiol in FST

As shown in Figure 2(b), both dosage groups with 10 mg/kg and 40 mg/kg Δ^3^,2-hydroxybakuchiol significantly reduced immobility time in FST. Moreover, this test showed that Δ^3^,2-hydroxybakuchiol at 40 mg/kg exhibited an effect similar to that of 2.5 mg/kg bupropion (*P >*0.05, 40 mg/kg Δ^3^,2-hydroxybakuchiol versus 2.5 mg/kg bupropion).

### 3.3. Effect of Δ^3^,2-Hydroxybakuchiol on Spontaneous Activity in OFT

**Figure 3:**
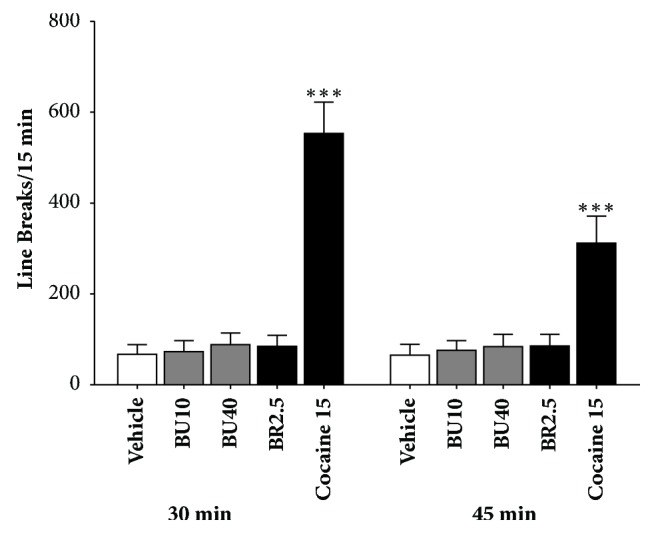
Δ^3^,2-Hydroxybakuchiol did not increase spontaneous movement in OFT. Balb/c mice were intraperitoneally injected with vehicle, 10 mg/kg Δ^3^,2-hydroxybakuchiol, 40 mg/kg Δ^3^,2-hydroxybakuchiol, 2.5 mg/kg bupropion, or 15 mg/kg cocaine, respectively (n = 10). Then mice were subjected to the OFT for 45-min. The line breaks between 15-30 min and 30-45min were recorded. The data are expressed as mean ±  SEM. Compared with vehicle, *∗∗∗P *< 0.005.

In OFT, spontaneous movement was measured after 30-min or 45-min treatment. As shown in Figure 3, at the two time points during which TST and FST were conducted, number of line breaks in mice with 15 mg/kg cocaine dramatically increased (*P <* 0.01, compared to vehicle). Unlike reference cocaine, this kind of stimulated spontaneous activity in groups of 10 and 40 mg/kg Δ^3^,2-hydroxybakuchiol, as well as 2.5 mg/kg bupropion, however, did not appear (*P >* 0.05, compared to vehicle control).

### 3.4. Effect of Δ^3^,2-Hydroxybakuchiol on Sucrose Preference in CUMS Rats

**Figure 4:**
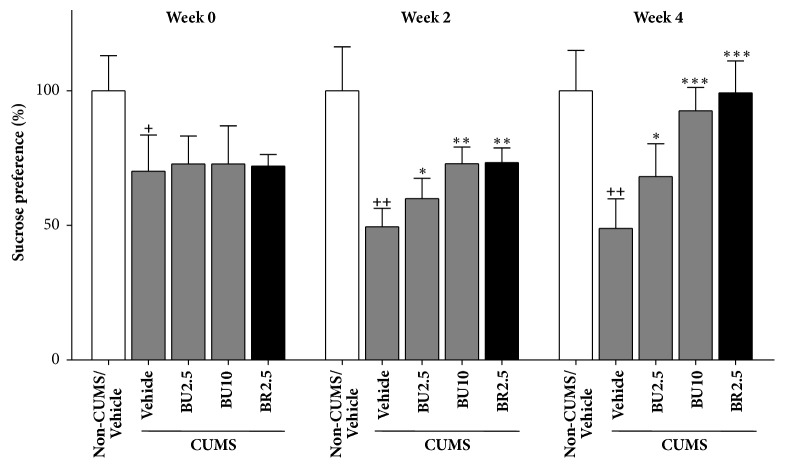
Δ^3^,2-Hydroxybakuchiol increased sucrose preference. Except for nonstressed group (Non-CUMS/Vehicle), Sprague-Dawley rats underwent CUMS procedure for 8 weeks (n = 10). All rats underwent SCT and daily intraperitoneal injection of vehicle, Δ^3^,2-hydroxybakuchiol, or bupropion with indicated dosages after the first 4-week stress procedure. Data was obtained before treatment (week 0) and after 2-week treatment and 4-week treatment. Values are expressed in the percentage of Non-CUMS/Vehicle group as mean ±  SEM. Compared with Non-CUMS/Vehicle group, +*P* < 0.05 and ++*P* < 0.01; compared with CUMS/Vehicle group, *∗P* < 0.05, *∗∗P* < 0.01, and *∗∗∗P* < 0.005.

Because disrupted reward processing is a core symptom of depressive disorder [13], the antidepressant effects of Δ^3^,2-hydroxybakuchiol on behaviours relating to rewards were investigated by evaluating sucrose preference of CUMS rats, which is usually detected by SCT for illustrating degree of anhedonia. As shown in Figure 4, after 4-week stress procedure prior to treatment (seen as week 0), CUMS/Vehicle group experiencing a four-week stress exhibited a significant decrease in sucrose consumption compared to that of Non-CUMS/Vehicle group (*P <* 0.05), whereas all CUMS treatment groups at week 0 did not present notable difference compared to CUMS/Vehicle group (*P* > 0.05). The result demonstrated that CUMS model was successfully established and grouping in week 0 met statistical uniformity. Significant difference of sucrose consumption between CUMS/Vehicle group and Non-CUMS/Vehicle group was also shown after administration for 2 weeks and 4 weeks (*P <* 0.01), representing continuous stress stimulation to rats. However, group with Δ^3^,2-hydroxybakuchiol at doses of 2.5 mg/kg or 10 mg/kg showed an increase in sucrose preference in CUMS rats on both 2-week and 4-week treatment (*P <* 0.05, compared to CUMS/Vehicle group). By the way, action produced by 10 mg/kg Δ^3^,2-hydroxybakuchiol was similar to that done by 2.5 mg/kg bupropion (*P > *0.05).

### 3.5. Inhibitory Effect of Δ^3^,2-Hydroxybakuchiol on Human DA/NE Transporters

**Figure 5:**
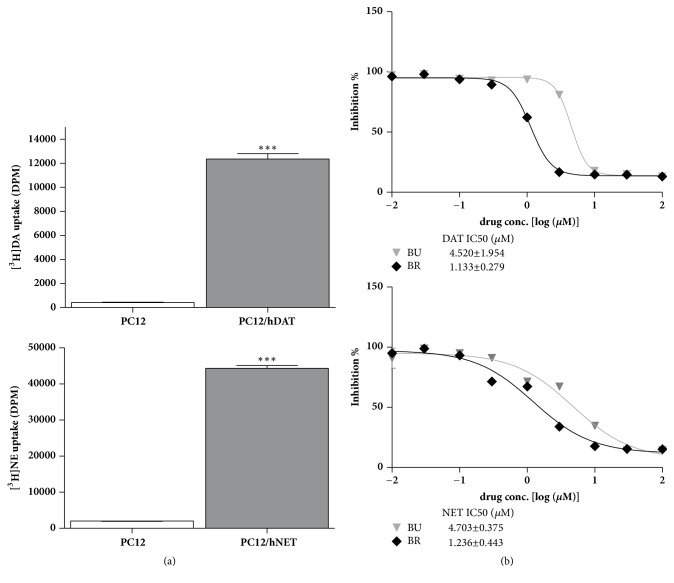
Inhibition of hDAT and hNET by Δ^3^,2-hydroxybakuchiol in PC12 cells overexpressing hDAT or hNET. (a) The validity of stable transporter-overexpressing cells was confirmed by comparing the isotope uptake between PC12-overexpressing cells and naive cells. (b) IC_50_ was calculated by the uptake inhibition under a serial of concentrations of Δ^3^,2-hydroxybakuchiol. Values are expressed as mean ± SEM. *∗∗∗P* < 0.005 versus naive cells.

Figure 5(a) showed a 20~30-fold enhancement of [^3^H]DA/NE uptake in PC12/hDAT or PC12/hNET cells relative to naive PC12, respectively, indicating a successful construction of DA/NE-transporter-overexpressed cell clones. Using these cell clones, IC_50_ values for Δ^3^,2-hydroxybakuchiol and reference bupropion in PC12/hDAT platform were 4.520±1.954 and 1.133±0.279 *μ*M, respectively; and IC_50_ values for Δ^3^,2-hydroxybakuchiol and reference bupropion in PC12/hNET platform were 4.703±0.375 and 1.236±0.443 *μ*M, respectively (Figure 5(b)). Despite a less potency, efficacy of Δ^3^,2-hydroxybakuchiol is similar to that of bupropion (Δ^3^,2-hydroxybakuchiol achieved maximal inhibition similar to this of bupropion).

### 3.6. Effect of Δ^3^,2-Hydroxybakuchiol on Monoamine in Microdialysate

**Figure 6:**
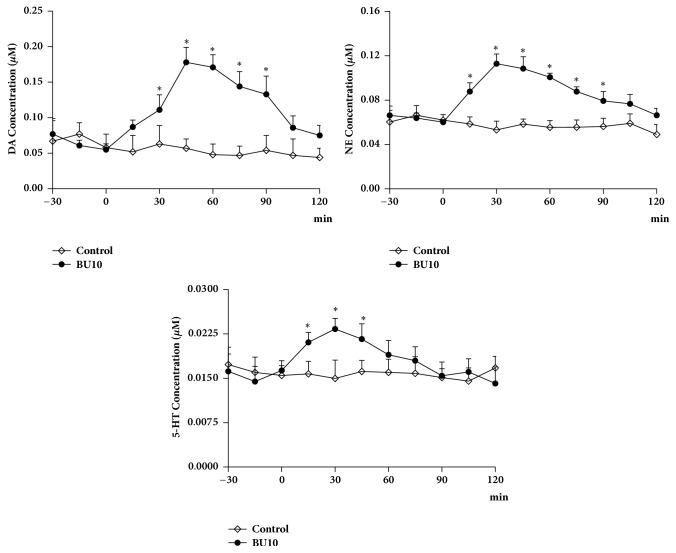
Δ^3^,2-Hydroxybakuchiol (BU, 10 mg/kg) retained extracellular concentration of monoamine neurotransmitters in rat striatum. After intraperitoneal injection of 10 mg/kg Δ^3^,2-hydroxybakuchiol (n = 3), dialysate fractions were collected at 15 min interval for continuous 120 min. The samples for basal concentration were collected in normal rats for n = 2. The levels of DA, NE, and 5-HT were detected by HPLC. Values are expressed as mean ± SEM. *∗P* < 0.05 versus control.

As shown in Figure 6, time-concentration curves indicated a significant effect of Δ^3^,2-hydroxybakuchiol on DA, NE, and 5-HT concentrations in microdialysate from striatum (*P <* 0.05, compared to vehicle) seen in several time points. Trend analysis revealed that vehicle control group did not produce any peak release of monoamine, whereas Δ^3^,2-hydroxybakuchiol treatment, as expected, led to continuously increased levels of these monoamine neurotransmitters and achieved maximums at about 30 ~45 min; then the upward trends turned downward and return to basal levels at approximately 90 ~120 min after drug administration. As for comparison of respective curve profile, degree of DA increase, which was seen to achieve nearly three times enhancement compared with control at peak time point, was generally higher than that of NA and 5-HE.

### 3.7. Anticarcinogenic Effect of Δ^3^,2-Hydroxybakuchiol

**Figure 7:**
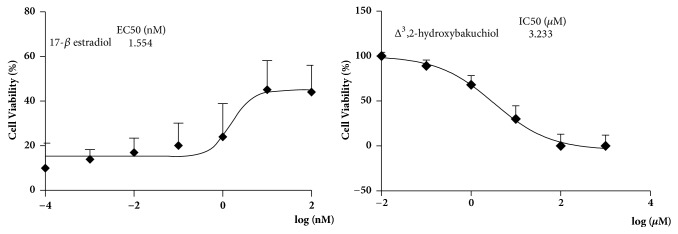
Δ^3^,2-Hydroxybakuchiol inhibited proliferation in MCF-7 cells. MCF-7 was treated with the indicated concentrations of Δ^3^,2-hydroxybakuchiol or 17-*β* estradiol for 72 h. Cell number was measured with MTT assay. IC_50_ or EC_50_ was calculated. Values are expressed as mean ±SEM.

Due to estrogenic effect of bakuchiol analog, carcinogenic or anticarcinogenic effect of Δ^3^,2-hydroxybakuchiol should be detected to evaluate its safety by tumourigenicity test upon malignant cell (MCF-7) line. As shown in Figure 7, after culturing for 72 h, Δ^3^,2-hydroxybakuchiol did not induce tumour cell proliferation and, unexpectedly, showed a mild anticarcinogenic effect with an EC_50_ value of 3.2 *μ*M for MCF inhibition; however, 17-*β* estradiol was shown to promote MCF-7 cell proliferation.

## 4. Discussion

Δ^3^,2-Hydroxybakuchiol, bakuchiol analog [8], is one of the major active compounds in* Psoralea corylifolia* (L.), a herb that is known to be one of the constituents in Chinese traditional medicine prescriptions used for depression treatment [14]. In our previous study, Δ^3^,2-hydroxybakuchiol decreased immobility in reserpinized-pretreated mice, indicating its antidepressive potential [8]. Thus, the objective of this study was to explore the potential antidepressant effects of Δ^3^,2-hydroxybakuchiol and to elucidate the action mechanism of antidepression both* in vivo* and* in vitro*.

Depression is characterized by a diversity of emotional and behavioural symptoms. These complex psychiatric and behavioural traits in human cannot be displayed or simulated with great accuracy by a single rodent model [15]. Evaluation of animal ethology and examination of behaviour indexes by using a series of animal models become important means for antidepressant drug research and development (R&D) [16]. Due to difficulty in simulation of human cognition and emotion only using one model, a combined use of multiple rodent models such as acute models (FST, TST) and chronic model (CUMS) in this study may be valid for antidepression evaluation.

FST and TST models are often used to evaluate behavioural despair, which is known to be one of the depressive endophenotypes. When encountering acute stress in FST and TST, individual ceases changing and controlling situations to present the negative view and their immobility time is the major index [17, 18]. Due to effectiveness and simplicity [19], the two tests were adopted and used for efficacy evaluation in this study. Our results (Figures 2(a) and 2(b)) showed that immobility time in TST and FST mice gradually decreased with increase in dosage of Δ^3^,2-hydroxybakuchiol, revealing a dose-dependent action tendency, which indicates that Δ^3^,2-hydroxybakuchiol can relieve behavioural despair caused by acute stress. Additionally, using OFT model, both 10 mg/kg and 40 mg/kg of Δ^3^,2-hydroxybakuchiol and 2.5 mg/kg bupropion did not statistically increase spontaneous movement of mice compared to reference cocaine group after 30-min or 40-min treatment, the two time points at which TST and FST were conducted (Figure 3). It is demonstrated that Δ^3^,2-hydroxybakuchiol cannot affect the spontaneous activity and that a false positive possibility attributable to movement stimulation like cocaine is excluded, suggestive of validity of Δ^3^,2-hydroxybakuchiol efficacy in both acute models.

In addition to acute models, chronic model of depression was adopted for activity evaluation. CUMS is a chronic and classic depression model in which animals encounter long-term stress, which is highly similar to major depression, a kind of persistent mental illness, and thus is suitable for more accurate evaluation of drug efficacy [20]. SCT in CUMS is a common test to evaluate anhedonia, another depressive endophenotype that presents inability to experience pleasure [21, 22]. In this study, after the first 4-week consecutive stress, all CUMS groups exhibited a significant decrease in sucrose consumption, indicative of a successfully established CUMS model. Increase of sucrose preference is often used to represent the improvement of anhedonia [21]. As shown in Figure 4, CUMS animals in group with 2.5 mg/kg or 10 mg/kg Δ^3^,2-hydroxybakuchiol showed significant increases of sugar consumption, and meanwhile the effect in 10 mg/kg dosage group was similar to that of 2.5 mg/kg bupropion after a 2-week or a 4-week treatment, demonstrating that Δ^3^,2-hydroxybakuchiol possesses a strong antidepressant activity in chronic stress model. The result obtained in CUMS is consistent with that in above-mentioned TST or FST model, as well as with reserpinized-pretreated model in our previous study [8]. These results shown in both acute and chronic models of depression provide credible evidences that support the antidepressant-like effects of Δ^3^,2-hydroxybakuchiol.

A large body of studies has demonstrated that monoaminergic transmitters modulate depressive symptoms and play important roles in response to stress [23]. The decline of NE, DA, and 5-HT in brain has been found in animal models of depression as well as in patients with depression and has been suggested as a major hypothesis in pathophysiology of depression [3, 24]. Clinical antidepressive drugs presently used are through a mechanism of increasing bioactivity of monoaminergic transmitters. The following study was conducted to explore whether antidepression action of Δ^3^,2-hydroxybakuchiol could be mediated by increasing concentrations of monoamine neurotransmitters using both* in vitro* and* in vivo* models. In our previous study, Δ^3^,2-hydroxybakuchiol presents potent inhibitions of rat DAT and NET and a mild inhibition of rat SERT in transporter-transgenic CHO cell line [8]. In neural cell line PC12 overexpressing human DAT or NET, isotope uptake studies showed that IC_50_ values of Δ^3^,2-hydroxybakuchiol for both transporters were similar to that of reference bupropion. These results are consistent with that in CHO platform overexpressing rat DAT and NET in our previous study [8]. It is further confirmed that Δ^3^,2-hydroxybakuchiol possesses a strong potency for human monoamine transporters. Monoamine transporter inhibition indicates that monoamine reuptake inhibition could be followed by increase in levels in synaptic cleft in brain.

As for this, we verified whether Δ^3^,2-hydroxybakuchiol could retain levels of monoamine neurotransmitters in synaptic cleft in brain using microdialysis and HPLC. Because striatum is the major brain region producing monoamine, levels of monoamine transmitter in striatum were detected to evaluate DA, NE, and 5-HT level. The microdialysis data exhibited that Δ^3^,2-hydroxybakuchiol strongly increased DA and NE levels and mildly increased 5-HT level in striatum, indicating that it is able to retain monoamine contents in synaptic cleft through which the antidepressive action could be generated. This result echoes the inhibitory actions on monoamine transporters* in vitro* [8] since inhibition of these transporters can increase the level of monoamine neurotransmitters in synaptic cleft in striatum. Some literatures have reported that NE and DA are associated with sleepiness [25] and fatigue [26]. Therefore, it is speculated that increase in NE and DA by Δ^3^,2-hydroxybakuchiol could ameliorate insomnia and fatigue and, thus, could be helpful in curing depression.

Antidepressants with low-toxicity still need to be considered [27]. Due to some similarity in oestrogen receptor agonism between 17-*β* estradiol, known to have breast cancer risk, and bakuchiol, an analog of Δ^3^,2-hydroxybakuchiol [28, 29], carcinogenic effect of Δ^3^,2-hydroxybakuchiol should be detected. Our result showed that Δ^3^,2-hydroxybakuchiol did not induce tumour cell proliferation, whereas it produced a mild anticarcinogenic effect, indicative of its safety. Additionally, toxicological experiments reveal a high dosage of i.p. LD_50_ (median lethal dose) (1101.8 mg/kg) for Δ^3^,2-hydroxybakuchiol in mice [30], further corroborating its safety.

## 5. Conclusion

In summary, Δ^3^,2-hydroxybakuchiol exhibits an antidepressant-like effect* in vivo*. This action is mediated possibly by inhibition of monoamine transporters and then increase of monoaminergic activity in brain. Δ^3^,2-Hydroxybakuchiol, with low-toxicity and high efficacy, may be a potential agent for depression therapy.

## Data Availability

All relevant data supporting the findings of this study are within the paper.

## Abbreviations

aCSF: Artificial cerebrospinal fluid
BR2.5: 2.5 mg/kg bupropion group
BU2.5: 2.5 mg/kg Δ^3^,2-hydroxybakuchiol group
BU10: 10 mg/kg Δ^3^,2-
BU40: 40 mg/kg Δ3,2-hydroxybakuchiol group
IC50: 50% inhibitory concentration
CUMS: Chronic unpredictable mild stress
Non-CUMS/Vehicle: No CUMS experience
DAT: Dopamine transporter
DMSO: Dimethyl sulfoxide
DPM: Disintegrations per minute
FST: Forced swim test
HBSS: Hank's balanced salt solution
HPLC: High performance liquid chromatography
i.p.: Intraperitoneally
LD_50_: Median lethal dose
MCF-7: Michigan Cancer Foundation-7
MTT: 3-(4,5-Dimethylthiazol-2-yl)-2,3-diphenyltetrazolium bromide
NET: Norepinephrine transporter
OD: Optical density
OFT: Open field test
PBS: Phosphate-buffered saline
SCT: Sucrose consumption test
SERT: Serotonin transporter
TST: Tail suspension test.

## Supplementary Materials

Supplementary Materials
* (1) Graphic Abstract*.** Experimental design diagram for evaluation of antidepressant-like effect of **Δ^3^,2-hydroxybakuchiol. This study includes two aspects: (1) effect study; (2) mechanism study. In effect study (left), depressive-like behaviours were ameliorated by Δ^3^,2-hydroxybakuchiol (BU) by using acute depression models, such as tail suspension test (TST) and forced swim test (FST), and chronic depression model such as chronic unpredictable mild stress (CUMS). In mechanism study (right), cellular monoamine uptake detection and microdialysis assay were used to confirm an inhibitory effect of BU on dopamine transporter and norepinephrine transporter (red/blue linear graph) and then an increase of monoamine levels in synaptic cleft (black linear graph), indicating that antidepressant-like effect of Δ^3^,2-hydroxybakuchiol may result from monoamine transporter inhibition.
