# Supplementary material for: Δ3,2-Hydroxybakuchiol Attenuates Depression in Multiple Rodent Models Possibly by Inhibition of Monoamine Transporters in Brain
Source: Evid Based Complement Alternat Med. 2018 Jun 20;2018:1325141. doi: 10.1155/2018/1325141 (PMC6031082; doi:10.1155/2018/1325141)
Supplement: Supplementary Materials — (1) Graphic Abstract. Experimental design diagram for evaluation of antidepressant-like effect of Δ3,2-hydroxybakuchiol. This study includes two aspects: (1) effect study; (2) mechanism study. In effect study (left), depressive-like behaviours were ameliorated by Δ3,2-hydroxybakuchiol (BU) by using acute depression models, such as tail suspension test (TST) and forced swim test (FST), and chronic depression model such as chronic unpredictable mild stress (CUMS). In mechanism study (right), cellular monoamine uptake detection and microdialysis assay were used to confirm an inhibitory effect of BU on dopamine transporter and norepinephrine transporter (red/blue linear graph) and then an increase of monoamine levels in synaptic cleft (black linear graph), indicating that antidepressant-like effect of Δ3,2-hydroxybakuchiol may result from monoamine transporter inhibition. [file 1325141.f1.pdf]

Supplementary Material

1. Graphic Abstract

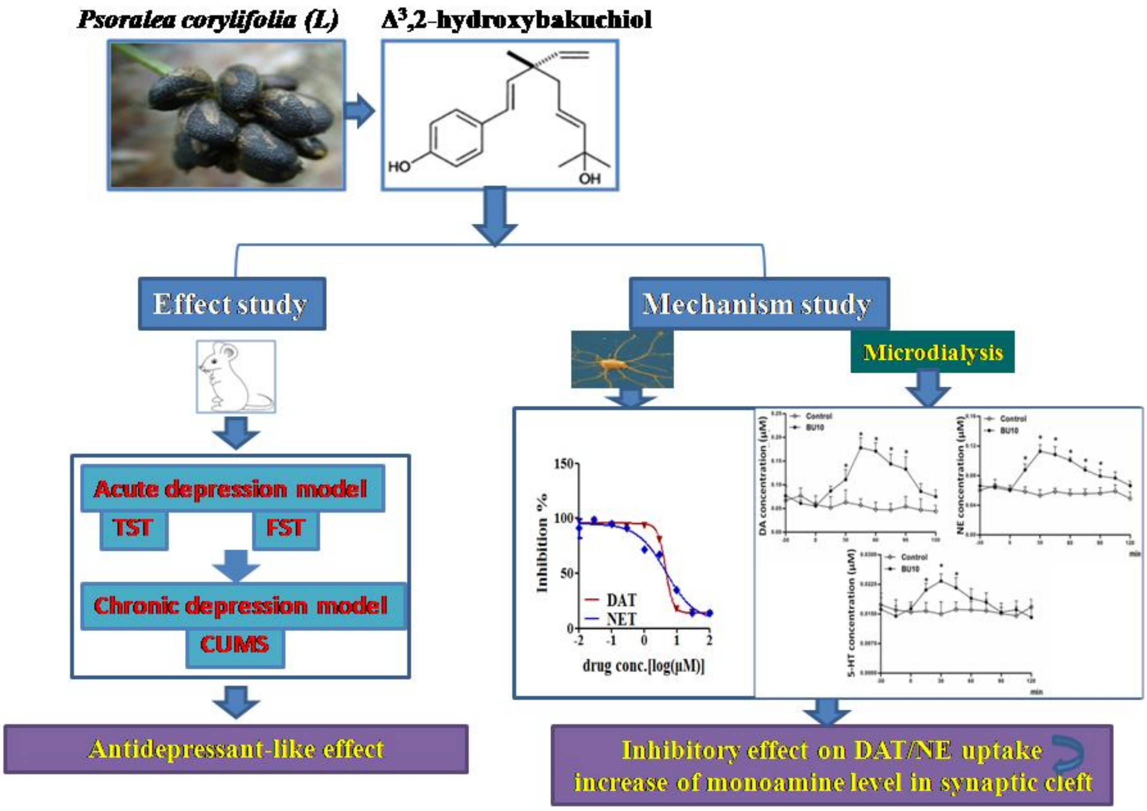

Experimental Design Diagram for Evaluation of Antidepressant-like Effect of Δ^3,2-Hydroxybakuchiol. This study includes two aspects: 1) effect study; 2) mechanism study. In effect study (left), depressive-like behaviours were ameliorated by Δ^3,2-hydroxybakuchiol (BU) by using acute depression models, such as tail suspension test (TST) and forced swim test (FST), and chronic depression model such as chronic unpredictable mild stress (CUMS). In mechanism study (right), cellular monoamine uptake detection and microdialysis assay were used to confirm an inhibitory effect of BU on dopamine transporter and norepinephrine transporter (red/blue linear graph) and then an increase of monoamine levels in synaptic cleft(black linear graph), indicating that antidepressant-like effect of Δ^3,2-hydroxybakuchiol may result from monoamine transporter inhibition.
